# The Controversial Clinicobiological Role of Breast Cancer Stem Cells

**DOI:** 10.1155/2008/492643

**Published:** 2009-03-19

**Authors:** Claudia Casarsa, Saro Oriana, Danila Coradini

**Affiliations:** ^1^Experimental Oncology Laboratory, Senology Center, Ambrosiana Clinic, Cesano Boscone, 20090 Milano, Italy; ^2^Surgery Department, Senology Center, Ambrosiana Clinic, Cesano Boscone, 20090 Milano, Italy

## Abstract

Breast cancer remains a leading cause of morbidity and mortality in women mainly because of the propensity of primary breast tumors to metastasize. Growing experimental evidence suggests that cancer stem cells (CSCs) may contribute to tumor progression and metastasis spread. However, despite the tremendous clinical potential of such cells and their possible therapeutic management, the real nature of CSCs remains to be elucidated. Starting from what is currently known about normal mammary stem/progenitor cells, to better define the cell that originates a tumor or is responsible for metastatic spread, this review will discuss experimental evidence of breast cancer stem cells and speculate about the clinical importance and implications of their evaluation.

## 1. Introduction

Despite significant advances in diagnosis and clinical management, breast cancer remains a leading cause of morbidity and mortality in women [1], mainly owing to the propensity of primary breast tumors to metastasize to regional and distant sites such as lymph nodes, lung, liver, bone, and brain [2]. When the primary tumor is detected and removed before metastasis occurs, prognosis could be good and the chance of disease-free survival is high. However, if cancer cells have already begun to disseminate from the primary tumor and spread to other organs, current therapeutic strategies largely depend on the use of systemic cytotoxic drugs that frequently result in severe side effects on the patient and, in many cases, do not yield long-term success.

This clinical scenario is further complicated by the fact that invasive breast cancers exhibit a wide range of morphological types, molecular profiles, and clinical behaviors. Not only there is a large variation in the nature of cell types between cancers, but even within a single tumor a significant heterogeneity in phenotype and genotype can be observed [3]. Based on growth patterns and cytological characteristics of the tumor cells, invasive breast cancers are categorized by the World Health Organization into 18 different histological subtypes, each of them associated with a diverse clinical behavior. In addition to morphology, invasive breast cancers can also be classified according to their proliferative potential (evaluated, e.g., by Ki67 expression) or the presence of such biological factors as hormone receptors (estrogen [ER] and progesterone [PgR]) or HER2/neu overexpression that are currently used in clinical practice to predict the prognosis and the response/resistance to cytotoxic and/or hormonal therapy [4–6].

Understanding the molecular causes of such a heterogeneity is therefore of paramount importance not only for the development of new therapeutic approaches, but also for a better knowledge of the biological bases of breast tumorigenesis and metastatic spread. To address these questions, over the past decade, scientists have used innovative technical strategies and approached new intriguing directions aimed to define the genetic and epigenetic profile of the single tumor and to better define the cell that originates a tumor or is responsible for metastatic spread. In particular, investigators focused their attention on the hypothesis that breast cancer may be a stem cell disease, arising from tissue stem/progenitor cells or driven by cells with stem-cell properties [7].

## 2. Isolation and Characterization of Mammary Stem/Progenitor Cells

In the adult, cell loss, associated with the physiological tissue turnover, is compensated by the activity of specific cells, termed stem cells, which are so defined by their ability to self-renew and to generate the entire repertoire of the differentiated cells composing a given tissue. Since 1983, the existence of such stem cells has also been postulated in the mammary gland to explain the cellular dynamics underlying morphological changes throughout a woman's life, particularly during and after pregnancy [8].

In general, the identification and purification of normal stem cells are difficult tasks because of the paucity of stem cells in the tissue of origin and to the lack of stemness-specific morphologic traits. Hence, animal models have been used, and the murine model, in particular, has supplied relevant data to improve our understanding of the cell biology of the mammary gland and to clarify the presence of stem/progenitor cell and differentiated cell compartments in the mammary gland [9, 10]. However, the information obtained from mice cannot be directly applied to humans (as in the case of murine exclusive cell surface markers) or may not be promptly translated to humans, as known in the hematopoietic field, where an opposite surface-antigen profile was observed in humans (CD34^+^CD38^−^) with respect to mice (CD34^−^CD38^+^). Several in vitro strategies have thus been developed to isolate and characterize human mammary stem/progenitor cells, based on the differential expression of some cell surface markers, the formation of mammospheres, and the use of fluorescent dyes.

### 2.1. Cell Surface Markers

The first experimental evidence of the existence of human mammary stem/progenitor cells was obtained by the in vitro isolation of multipotent epithelial cells, from normal human adult breast, according to their different expression of MUC-1 glycoprotein, CALLA/CD10, and epithelial-specific antigen (ESA). Using these markers, two epithelial-cell progenitor populations were distinguished, corresponding to the two components of normal mammary gland (*myoepithelial cells*, forming the basal layer of ducts, and *epithelial cells*, lining the lumen of ducts and forming the alveoli) [11–13]. Subsequently, Gudjonsson et al. [13], starting from the previous findings [14], provided in vivo evidence of the morphogenic potential of the MUC-1^−^/ESA^+^ subpopulation, inoculating these cells subcutaneously in nude mice after pre-embedding them in a mixture of collagen gel and matrigel.

### 2.2. Mammosphere Formation

To identify a human mammary stem/progenitor-cell subpopulation, Dontu et al. [15] adopted a strategy similar to that employed for primary neural cells and based on the formation of floating spherical colonies used to define and measure stem cell-like behavior. In fact, contrary to the dogma that epithelial cell survival is anchorage-dependent, single cell suspensions of human mammary epithelial cells, obtained by mechanic/enzymatic dissociation, surprisingly survived in suspension and generated floating spherical colonies, termed nonadherent *mammospheres* [15]. The mammospheres contained numerous undifferentiated cells that, once isolated from the cluster, were able to generate new multilineage colonies, when cultured under differentiating conditions, and, in 3D culture, to reconstitute a functional mammary gland. However, the intrinsic dynamics of such cytospheres, as conventionally assayed, introduces several confounders, as reported in a recent paper by Singec et al. [16] who underlined the need to use more accurate conditions for assessing the clonality, number, and fate of stem cells. Although sphere formation may represent a useful culturing tool, it is not specific to stem cell characterization; any dividing cell from virtually any tissue will form floating cell clusters, when cultured in a serum-free medium and on a nonadherent substrate, owing to a predominant intercellular adhesiveness. Spontaneous sphere fusion may occur in normal as well as in neoplastic sphere cultures. Furthermore, in agreement with Singec et al. [16], we have observed that the mammospheres, supposedly rich in multilineage progenitors, have a very short life span (about 3-4 weeks), making it difficult to define the cells composing them as real mammary stem cells, which are long-lived by definition. On account of all these criticisms, this experimental approach, based on the ability of the supposed mammary stem cells to generate clonal mammospheres under anchorage-independent culture conditions, has been defined “a surrogate stem cell assay” [17].

### 2.3. Fluorescence Methods

To overcome the challenge represented by the limited availability of stemness markers and to take advantage of the ability of stem cells to extrude dyes, for example, Hoechst-33342 DNA-binding dye or rhodamine because the overexpression of some membrane transporter proteins, such as P-glycoproteins or breast cancer resistance proteins (BCRPs) [18, 19], fluorescent dyes have been used to identify and isolate by flow cytometry a small fraction of cells supposed to be stem progenitors [20]. This cell fraction, which amounts to around 0.2% of the total population, has been called *side population* (SP).

However, several criticisms to such a sorting technique have been raised in the recent years principally concerning the toxicity of dyes used in the analysis [21] and the high assay variability associated with technical modifications required for each cell population under study; these limitations hamper the comparison of results obtained from different studies and affect cell selection for in vitro and in vivo growth experiments. In addition, recent findings from two teratocarcinoma cell lines indicate that Hoechst treatment, as performed during staining for SP analysis, can affect cell differentiation, suggesting other potential complications in the interpretation of data [22].

Recently, another approach, based on aldehyde dehydrogenase (ALDH) activity, has been proposed as a promising alternative to identify and characterize the human mammary stem/progenitor component in the mammary gland [23]. ALDH, a detoxifying enzyme responsible for the oxidation of intracellular aldehydes, is a putative candidate marker of stemness, since it is highly expressed in hematopoietic and neuronal stem/progenitor cells. Its presence can be evaluated by ALDEFLUOR kit: brightly fluorescent ALDH-expressing cells are easily detected by flow cytometry in the green fluorescence channel. Using such an approach, Ginestier et al. [23] showed that ALDH-positive cells formed mammospheres with high efficiency (10 ± 3.5%) when put into 96-well plates (1 cell/well), and displayed stem-like properties in terms of bilineage differentiation in vitro and outgrowth potential when inoculated in the mammary fat pad of humanized mice. However, even though the findings indicate that only ALDH-positive cells had phenotypic and functional characteristics of mammary stem cells, immunostaining of tissue sections using a monoclonal antibody against the first isoform of ALDH (ALDH1) did not detect any overlapping expression of several markers (e.g., CK5/6 and CK14), previously associated with undifferentiated mammary epithelial cells, probably owing to the scarcity of this population. Analysis performed on mammosphere sections have shown that ALDH1-positive cells represented approximately 5% of the total cell population and expressed CK5/6 or CK14, supporting the hypothesis that ALDH1-positive cells represent the stem/progenitor population [13, 15].

Unfortunately, as highlighted by these inconclusive results, the efforts to purify adult stem cells from the human mammary gland have so far been hampered, on the one hand, by the lack of cell surface markers specific to undifferentiated or differentiated mammary cells and, on the other hand, by the lack of suitable in vivo assays for testing stem cell properties, with the consequence that human breast stem cells have not yet been extensively characterized.

## 3. Cancer Propagation Models

**Figure 1:**
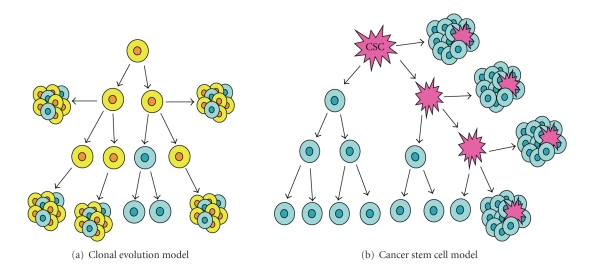
*Models of heterogeneity in solid cancer cells*. (a) The *clonal evolution model* assumes that every cell in a tumor is potentially tumor-initiating. Progression is governed by rare stochastic events operating in all cells. Cells with mutations (*yellow*) that acquire growth advantage will dominate over all other cells in the tumor and will originate a new clone containing cells characterized by a different phenotype and having different proliferative potentials; in a clonogenicity or tumorigenicity assay, some of these cells (*blue*) would have a low probability of exhibiting this potential. (b) The *cancer stem cell model* states that a particular subset of tumor cells with stem cell-like properties, called “cancer stem cell” (CSC) (pink), drives tumor initiation, progression, and recurrence. CSCs are able to self-renew indefinitely and to differentiate, leading to the production of all cell types (blue) that make up the rest of the tumor. In clonogenic assays, CSCs have the potential to proliferate extensively and can form new tumors on transplantation.

To explain why not every cell within a tumor is capable of maintaining and/or reinitiating tumor growth, two models of heterogeneity in solid cancer have been proposed: the *clonal evolution model* (Figure 1(a)) and the *cancer stem cell* (CSC) *model* (Figure 1(b)) [24–26].

The two main aspects of the *clonal evolution model,* first proposed by Nowell in 1976 [27], are (1) diversity within the tumor due to genetic instability and (2) selection of the cells with the most advantageous phenotype. In this respect, stem or differentiated cell characteristics (including self-renewing capacity) are just simple phenotypes and, as such, can change. According to this model, any cancer cell can potentially become invasive and cause metastasis or become resistant to therapies and cause recurrence.

The *cancer stem cell* (CSC) *model* (Figure 1(b)) states that a particular subset of tumor cells with stem cell-like properties, called “cancer stem cells”, drives tumor initiation, progression, and recurrence. Since CSCs are widely believed to arise from normal stem or progenitor cells, the identification of stem cells in a tissue is of paramount importance to understand how a tumor arises. By definition, CSCs have the ability to self-renew indefinitely and to differentiate, which leads to the production of all cell types composing a tumor, both tumorigenic and nontumorigenic cells. But the latter lack the unlimited self-renewing capacity and the ability to reproduce the phenotypically diverse cell populations that make up the tumor bulk [28, 29]. Therefore, this model suggests that the presence of such rare tumor-initiating cells, in the heterogeneous mix of cells composing a tumor, is essential for neoplastic progression and metastatic spread [29, 30]. While the *CSC model* of carcinogenesis was described in the context of systemic malignancies as long ago as in the 1930s, only recently has it been extended to solid tumors, and during the last decade, several studies have provided evidence that CSCs may also exist in solid tumors including brain [31], lung [32], prostate [33], colon [34], liver [35], pancreas [36], and breast [37] carcinomas, as well as melanoma [38].

In breast tumorigenesis, the *CSC model* also seems to be supported by clinical observations indicating that, despite the fact that breast cancer patients may have hundreds or thousands of single disseminated cancer cells detectable in their bloodstream, only a very small percentage of cells progresses to form overt macroscopic metastases [39], and that metastatic tumors tend to reproduce a heterogeneity similar to the primary tumor [40]. Since CSCs have been supposed to be responsible for the chemo- and radioresistance observed in several solid tumors [41, 42], the *CSC model* could explain such a finding with the ability of CSCs to escape cytotoxic drugs, via a high expression of specific drug transporter proteins, and to resist radiotherapy by increasing DNA repair activity.

Unfortunately, the definitive proof of the cellular origin of CSCs—they may arise either from normal resident stem cells within the tissue bearing the malignancy or from transformed progenitor cells that acquire the stem cell ability of self-renewal—remains elusive and is a topic of intense debate as well as experimental investigation. In fact, if these cells arise from normal stem cells, then cancer cells could take advantage of the existing regulatory self-renewal pathways of stem cells. On the other hand, if these cells arise from mature, differentiated cells, multiple oncogenic mutations, affecting differentiation and self-renewal pathways are required for a cell to become tumorigenic and metastatic [43, 44]. It can be argued that mature cells have a very limited life span, and thus it is unlikely that all the necessary mutations could occur during the relatively short life of these cells. In contrast, the unlimited self-renewing capacity of normal stem cells could enable them to accumulate the necessary mutations, despite the apparent paradox of the stem cell dogma, according to which a stem cell maintains its DNA constant through symmetric division [26, 45]. Thus, whereas the *CSC model* is highly hierarchical with a unique self-renewing cell type at the apex, the *clonal evolution model* attributes much of the intratumor variation to subclonal differences in mutational profile and implies that all cells, except the terminally differentiated ones, may have self-renewal capacity. Nevertheless, *clonal evolution* and *CSC models* share some aspects: in both, for instance, the tumor arises from a single cell that has acquired multiple mutations and has gained unlimited proliferative potential. This suggests that tumor heterogeneity could be explained by a new version of the clonal evolution model that incorporates some features of the CSC hypothesis [26].

**Figure 2:**
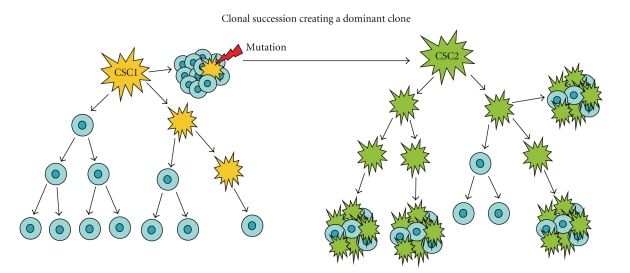
*Mixed model for the nature of sustained tumor growth*. The tumor is originally driven by rare cells of one phenotype (CSC1, yellow), which may have stem/progenitor cell origin and give rise to the tumor bulk by producing terminally differentiated cells (blue). Subsequent mutations enhancing self-renewing capacity create a dominant subclone that is phenotypically different (CSC2, green) and more aggressive. If the CSC2 subclone does not display “stem-like” cell properties, such a subset will not be able to initiate tumors with a high frequency [30].

A more intriguing model to explain the nature of sustained tumor growth is now emerging from the *CSC model.* According to this new model (Figure 2), tumors could originally be driven by rare CSCs (CSC1). Subsequent mutations, enhancing self-renewing capacity, could create a dominant, more aggressive subclone (CSC2), with a phenotypical aspect distinct from the original CSC. However, if the CSC2 subclone does not display “stem-like” properties, it should not be able to initiate tumors with a high frequency [46].

**Figure 3:**
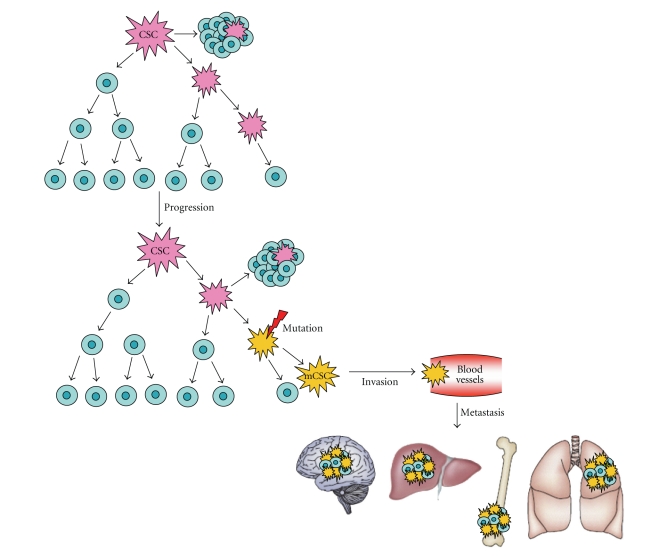
*Metastatic evolution*. Genetic and epigenetic mechanisms may cause the generation of a self-renewing metastatic cancer stem cell subclone (mCSC, yellow), phenotypically different from the CSC that is driving tumorigenesis (pink). Through a series of invasive processes, mCSC enters blood vessels and colonizes distant organs according to the seed and soil hypothesis. Blue cells represent bulk tumor cells [47].

Although metastasis is the predominant cause of lethality in breast cancer patients, metastatic spread is a highly inefficient process, since very few cells successfully colonize distant sites. A possible explanation of this inefficiency is provided by the *CSC model,* according to which genetic and epigenetic mechanisms may generate, within the primary tumor, a self-renewing metastatic cancer stem cell (mCSC) characterized by an immunophenotype different from the CSC that is driving tumorigenesis (Figure 3). This is suggested by the observation that in metastatic sites, some cell subpopulations with self-renewing features display a cell surface marker profile different from the CSC that originated the primary tumor [47]. Through a series of invasive processes, this new mCSC subclone could enter blood vessels and colonize distant organs according to the “seed and soil” hypothesis.

## 4. Isolation and Characterization of Breast Cancer Stem Cells

On account of accumulating evidence and according to the *CSC model* that assumes a tissue stem/progenitor cell as the origin of a tumor, some methods adopted to isolate and cultivate normal mammary stem/progenitor cells have also been applied to breast cancer tissue. However, there may be biases, inherent in the techniques applied, that can affect experiment reproducibility. In fact, some technical approaches, including the strong enzymatic digestion of breast tissue necessary to disaggregate the connective tissue surrounding the mammary gland, can cause damage to cells or loss of some particular surface markers, hampering the identification of unique markers for the putative CSCs (authors' unpublished data). Furthermore, since cell recovery from solid tissue is usually low (rarely exceeding 10%), the samples obtained may not be representative of the original lesion, owing to the rare presence of CSCs in the tumor mass, actual CSCs may be lost, while other cells may be mistakenly identified as CSCs [26].

The first experimental clues about the existence of putative CSCs came from the observation that, even when using immortalized cancer cell lines, large numbers of cells (in the range 10^5^–10^6^) must be injected into experimental animals to initiate a tumor and, in spite of that, only a very small proportion of these cells will go on to form metastases [48]. Additional experimental studies showed that while early steps in hematogenous metastasis (intravasation, survival, arrest, and extravasation) can be remarkably efficient, with over 80% of cells successfully completing the metastatic process at this point, only a small subset of these cells (i.e., about 2%, depending on the experimental model) can initiate growth as micrometastases and that an even smaller subset (i.e., about 0.02%, depending on the experimental model) is able to persist and grow into macroscopic tumors. This suggests that the initial growth of micrometastases represents the critical decision-making stage.

### 4.1. Surface Markers

After the identification of stemness specific markers in hematopoietic tumors, considerable progress has also been made in the elucidation of the biological properties of breast cancer stem cells. Al-Hajj et al. [37] first demonstrated the presence of a cell subpopulation displaying stem cell properties, characterized by the cell surface marker profile CD44^+^/CD24^low^/lin^−^, in solid tissues and in pleural effusions of patients with advanced-stage metastatic breast cancer. This phenotype displayed a 10- to 50-fold increase in the ability to form tumors in NOD/SCID mice over unfractionated tumor cells. In addition, the authors demonstrated that cells with such a specific cell surface antigen profile could successfully and efficiently grow as tumor xenografts in immunodeficient mice; the highest capacity to form tumors was observed after injection of 200 cells with the ESA^+^/CD44^+^/CD24^low^/lin^−^ phenotype.

However, the heterogeneous expression patterns of ESA, CD44, or CD24, observed by FACS analysis in secondary lesions, support the hypothesis that the CD44^+^/CD24^low^/lin^−^ profile could be the marker of the putative breast CSC phenotype, since it recapitulates the heterogeneous complexity of the tumors from which it has been isolated. Such a hypothesis could be corroborated by a study in which Ince et al. [49] observed the presence of two different populations of mammary epithelial cells in the tumor of a single patient. Only one population was myoepithelial-like and was able to give rise to tumors with heterogeneous histology and to be tumorigenic when injected into mammary fat pads of immunodeficient mice.

### 4.2. Fluorescence Methods

Similar to normal breast stem cells, CSCs have also been investigated according to their expression of aldehyde dehydrogenase (ALDH) activity. Ginestier et al. [23] analyzed the tumorigenicity of ALDEFLUOR-positive populations isolated from two metastatic and two primary invasive ductal carcinomas (three triple negative tumors—ER-negative, PR-negative, and HER2-negative—and one ER-positive, PR-positive, and HER2-negative tumor) that were transplanted into the humanized cleared fat pad of immunodeficient mice, immediately after surgery with no previous cultivation. To test tumorigenicity, cell sorting was performed at early passages in animals, in order to minimize the variability introduced by the xenotransplant model. ALDEFLUOR-positive cells represented 3% to 10% of the total cell population, and 500 positive cells were able to generate a tumor in as few as 40 days. Significantly, the concomitant presence of the ALDEFLUOR-positive phenotype and of the previously described breast CSC phenotype (CD44^+^/CD24^−/low^) was observed in a small cell fraction of the three triple-negative tumors (range 0.08–1.16%), whereas tumor cells generated from one metastatic tumor (pleural effusion) showed a high percentage of overlapping cell fraction (1.16%) that gave rise to outgrowth from as few as 20 cells. Conversely, ALDEFLUOR-negative cells, though bearing the CD44^+^/CD24^−/low^ phenotype, were not tumorigenic, even when 50 000 cells/fat pad were implanted. This suggests that the concomitant presence of ALDEFLUOR-positive and CD44^+^/CD24^−/low^ phenotypes may characterize progenitor cells with proliferative potential. When assessing the potential use of ALDH1 to detect malignant mammary stem/progenitor cells in situ on breast cancer tissue sections, Ginestier et al. [23] found that ALDH1 expression correlated with the histoclinical parameters, suggesting the use of this marker as a powerful predictor of poor clinical outcome. So far, therefore, the ALDEFLUOR assay, overcoming the limited availability of CSC-specific surface markers, seems to represent the pivotal tool for the isolation of cell populations with high tumor-initiating capability or cell populations with stem-like properties in normal tissue, allowing the identification of stem/progenitor cells involved in normal mammary development and may be in tumor transformation.

As regards the use of Hoechst 33342 to detect the side population and to identify CSCs, thus bypassing the lack of universally accepted surface-antigen markers, several limitations are emerging in addition to the technical criticisms described for the methods of normal mammary stem cell isolation; they include the low cell recovery from tumor tissue that does not reflect the entire cohort of cancer cells, and the toxicity of the dye that precludes its use for functional CSC assays in vitro and in vivo [50].

### 4.3. Self-Renewal Pathways

Since another trait shared by normal stem cells and CSCs is the ability to self-renew, the deregulation of key pathways involved in such a pivotal cellular function has been presumed to be implicated in breast carcinogenesis and more thoroughly investigated. Experimental evidence indicates that carcinogenesis in the mammary gland, and in other solid organs, might result in the transformation of stem and/or progenitor cells because of the deregulation of self-renewal pathways, including Notch, Wnt, Hedgehog, and the transcription factor Bmi1 [51] and suggests that the targeting of self-renewal pathways might provide a specific approach to eradicate CSCs [52]. However, in developing and testing compounds against putative CSCs, several uncertainties must be faced and elucidated, first of all the possible instability of CSCs that could hamper specific cell targeting.

## 5. Limits to the Xenograft Approach

There are several limitations to the use of xenograft assays as proof of “stemness”. The most relevant is that tumor growth and stem cell phenotype are not only determined by intrinsic characteristics of tumor cells but are also influenced by the microenvironment in which cells grow. In this respect, the heterogeneity found in animal experimental models does not prove that normal or malignant stem cells undergo asymmetric division but just reflects a change in cell surface antigen expression induced by environmental conditions. Simply injecting tumor cells into mice without measuring the time of latency required to form a palpable tumor mass may induce to draw wrong conclusions about their absolute tumorigenic potential, which may be influenced by environmental conditions. Another important limitation to CSC investigation is the efficiency of thein vivo model used. It is well known that xenograft is less efficient than syngeneic transplant because of the presence of animal growth factors that could interact with their equivalent human receptors and provide confounding stimuli for the transplanted human stem/progenitor cells. In a recent paper, Kelly et al. [53], challenging the CSC hypothesis, proposed that xenograft may select a dominant clone capable of surviving and maintaining tumor outgrowth in a foreign environment, and stressed the importance of performing a tumorigenic assay using cells sorted from the patient's tumor to avoid cell variability and the selection of a dominant cell subpopulation, after several serial passages in animals, whereas the majority of cells die owing to the lack of appropriate supporting factors.

As regards the mammary gland, in order to study human breast carcinogenesis, it is central to establish a model system that more accurately recapitulates normal breast epithelial development in rodents. For cancer cells, such a system should also correlate with the clinical behavior of the source tumor in patients. However, the inability of human breast epithelial cells to colonize mouse mammary fat pads represents a constant problem.

The importance of both species- and tissue-specific influences has been highlighted in the studies by Kuperwasser et al. [54], which indicated that, although outgrowths can be generated in the murine humanized mammary fat pads, the repopulating frequency by normal breast stem cells remains relatively low. This suggests that the expression of some markers of the inoculated cells could be influenced by circulating or locally produced animal-specific factors. For example, estrogen has been found to profoundly affect the growth of ER-negative breast cancer cells because circulating mouse estrogens led to recruitment of bone marrow-derived stromal cells and promoted the growth of tumors in virgin mice [55].

However, despite these limitations, xenograft models still represent an essential tool for in vivo carcinogenesis studies. Meanwhile, the great challenge in stem cell investigation will be the standardization of an orthotopic model in which the whole tumor bulk could arise from a single definitively characterized human breast CSC.

## 6. Issues Concerning Established Breast Cancer Cell Lines

Despite the intriguing results so far obtained, the use of established breast cancer cell lines as experimental models to collect data regarding CSCs is not without pitfalls, the main one being the attempt to apply stem-cell concepts to breast cell lines. Established cell lines, in fact, are cultivated in artificial conditions (depending on the experimental model) for many generations, with the risk that the unavoidable selection induced by the serum media blurs the distinction between tumorigenic and nontumorigenic clones. For example, in vitro culture conditions, such as growth with or without serum medium, could contribute to the functional differences found between non-CSCs and CSCs, including a diverse proliferative activity.

It is unlikely that the so-called “cancer stem cells” derived from established cell lines are the stem cells that make up a tumor. It is more likely that the “stem cell component” indicated as responsible for maintaining the line is in reality only a subpopulation of cells having a high-proliferative rate and being able to form clonal aggregates in the presence of additional techniques, for example, retroviral marking [16]. Therefore, extending the CSC concept to cell lines could be misleading as is the case with the results reported by Sheridan et al. [56]. In a series of established breast cancer cell lines (MDA-MB-231, TMD-436, Hs578T, SUM1315, HBL-100, and MDA-MB-468), the authors observed a high percentage (>30%) of CD44^+^/CD24^−/low^ cells which were highly efficient in initiating a tumor in experimental settings, and concluded that those cell lines were composed mainly of cancer stem cells. However, since the CD44^+^/CD24^−/low^ phenotype has been shown to be insufficient to confer stem-like properties [23], such an enrichment in CD44^+^/CD24^−/low^ phenotype in established cell lines could be hypothesized to be a purification marker without functional implications. Therefore, it is crucial to demonstrate that such a subpopulation, contained in established breast cancer cell lines and displaying a putative stem-like phenotype, has the real functional characteristics of CSCs: self-renewal and differentiation capability. Awaiting these confirmations as well as more standardized protocols for the isolation and expansion of CSCs from tissue, the established breast cancer cell line model still remains a useful experimental tool to test drugs, radioresistance, and antibodies against the surface markers associated with the putative stem-like phenotype.

## 7. Microenvironment and Stem Cell Niche

Despite the extensive use of the in vivo model, in which human tumor cells are injected into immunodeficient mice, significant challenges are pending in experimental settings with CSCs, mostly related to the biological and technical complexities associated with identifying, quantifying, and longitudinally monitoring CSCs in a complex in vivo environment. In particular, as already mentioned, xenograft may fail to reveal possible tumor growth-sustaining cells because the heterologous microenvironment precludes essential interactions with support cells. As recently shown in an elegant study by Naumov et al. [57], many established human tumor cell lines, which had previously been described as “nontumorigenic” (including the breast cancer cell line MDA-MB-436), were potentially tumorigenic but dormant. In fact, when these cells were injected into animals and allowed to grow for a much longer period of time than a normal tumor growth assay (up to 12 months), spontaneous tumors eventually began to develop after an initial dormancy period and a switch to an angiogenic phenotype. Therefore, this study provides a conceptual framework to approach the problem that a supportive microenvironment is required for tumor outgrowth in CSCs-involving studies.

In adults, normal stem cells reside in a physiologically unique microenvironment called stem cell *niche*. This niche, mainly composed of fibroblasts and myoepithelial cells, provides a physical anchoring site for stem cells via adhesion molecules linking the stem cells to the extracellular matrix. Support cells act as a hub in orientating dividing stem cells to hold one daughter cell in the niche, while the other one exits the niche and undergoes transit amplification followed by differentiation [58, 59]. Under normal physiological conditions, the niche provides fine control over cell proliferation, typically balancing proliferation and apoptosis through paracrine factors, so that the stem cell population remains undifferentiated and maintains a constant size [60, 61]. The effectors mediating heterotypic cell interactions within the niche include a number of soluble factors and cell surface receptors. Interestingly, some of these molecules, including Wnt, Notch, TGF-*β*, bone morphogenetic proteins (BMPs), and others [62–65], are known to be involved in tumor development, and emerging data support the idea that a “cancer stem cell niche” could also exist, and that interactions with such a tumor niche may sustain a self-renewing population of tumor cells [66]. Alterations affecting stromal cells, such as local modifications of tissue homeostasis induced by chronic inflammation, have been shown to promote formation of epithelial tumors, and very recent papers have described the generation of pluripotent stem cells from fibroblasts. This evidence supports the possibility that cancer may arise from just a few mutations in resident tissue stem/progenitor cells or even differentiated cells leading to a stem-like phenotype [67, 68]. Indeed, if signaling pathways are dysregulated, the niche may be converted into a microenvironment that favors uncontrolled proliferation and expansion of an altered stem cell population. Similarly, a cancer stem cell could be hypothesized to remain dormant in a metastatic site until activated by abnormal signaling from the microenvironment [66].

Currently, evidence of an anatomically and/or physiologically specialized environment that constitutes a true CSC niche is scarce, and the identity of a stem cell niche within the mouse mammary gland has not been defined. Conversely, a putative stem cell niche which gives rise to at least three lineage-restricted cell types outside the stem cell zone has been identified in the adult human breast [28]. The most likely location of the niche in the mature gland could be the ducts, where in situ analysis identified a narrow region of quiescent cells that were stained for putative stem cell markers including chondroitin sulphate, K6a, CK15, and SSEA-4 [69]. However, the data published so far do not exclude the possible existence of other niches or models of niche. In particular, the model of the estrogen-driven stem cell niche consists of three different cell types: the ER-positive sensor cells, the EGFR-positive stromal cells, and the ER-negative stem cells. All of them could remain quiescent until they are switched on by estrogens. In response to estrogens, the ER-positive sensor cells synthesize and secrete amphiregulin that activates EGFR-positive stromal cells which in turn activate ER-negative stem cells [70], although the identity of the stromal factors interacting with the epithelial components of the stem cell niche remains to be revealed. In addition, the similarities between stem cell niches in different tissues remain poorly understood, in particular whether tissue-specific stem cells can be regulated by stem cell niches in other organs or whether vice versa ectopic mesenchymal stem cells may colonize a breast niche and influence its behavior. Studies by Hochedlinger et al. [71] support the notion that a malignant genome can be reprogrammed to exhibit a normal-like phenotype when transferred into a new biological context, whereas Blanpain et al. [72] have reported that epithelial stem cells are able to generate their own microenvironment. Tumor cells also have the well-established ability to interact with their surrounding environment and to influence it profoundly; examples are neoangiogenesis, recruitment of immune cells, and modification of tissue architecture. All these findings have important and provocative implications for understanding metastatic growth in secondary sites.

## 8. CSCs and Metastasis

Since the majority of breast cancer deaths occur as a result of metastatic disease rather than from the effects of the primary tumor, one of the biggest challenges is the identification, as early as possible, of patients harboring metastatic cells. In fact, the persistence of disease at a low or undetectable level (the so-called “minimal residual disease”) is a common feature of breast cancer as supported by autoptic findings [73] as well as by the accumulating evidence that breast cancer patients, even with no indication of metastatic spread by current clinical parameters, have individual tumor cells in their blood [74, 75]. Several studies have shown that detection of isolated tumor cells in the bone marrow is an independent prognostic factor. However, even though approximately 30% of breast cancer patients may have micrometastatic disease in their bone marrow at the onset, only 30–50% of them will go on to develop clinically evident metastases within 5 years [76, 77].

The presence of such cells in the bone marrow is particularly interesting since bone represents one of the most common sites for breast cancer metastasis, together with regional lymph nodes, lung, liver, and brain, all of which may represent putative nichesfor disseminating tumor cells according to the hypothesis that cancer cells can arrest and grow in favorite metastatic sites. This “seed and soil” theory, first proposed in 1889 by Paget [78], predicts that a cancer cell (the “seed”) can survive in and colonize only secondary sites (the “soil”) that produce growth factors capable of significantly influencing cell behavior [79, 80], and has largely withstood the test of time [81]. In the case of breast cancer, disseminated carcinoma cells are detectable in the bone marrow [82], and recent findings indicate that most cancer cells found in the bone marrow have a breast CSC phenotype [83].

However, recent experimental observations demonstrated a direct involvement of the bone marrow-derived cells in the development of human epithelial tumors, suggesting that CSCs may originate from bone marrow-derived cells [84]. Furthermore, a very recent paper from Mylona et al. [85] indicated that, in clinical breast cancer tissues, the CD44^+^/CD24^−/low^ phenotype (i.e., the phenotype experimentally associated with stemness) had no significant correlation with clinical outcome. This is in agreement with a previous paper by Abraham et al. [86] that found no correlation between CD44^+^/CD24^−/low^ tumor cell prevalence and tumor progression, in terms of event-free and overall survival. Conversely and quite surprisingly, the prevalence of CD44^−^/CD24^+^ tumor cells was found to exert an unfavorable impact on patients' relapse-free and overall survival. This suggests that, although CD44^+^/CD24^−/low^ breast tumor cells may be highly efficient in initiating tumors in animal experimental models, in patients, these cells could be associated with the development of distant metastasis, particularly bone metastases, rather than with clinical outcome. Therefore, CD44^+^/CD24^−/low^ tumor cells could be a subclass of tumorigenic cells characterized by a great metastatic potential, maybe due to the role of CD44 as a homing receptor for distant tissue compartments.

## 9. Clinical Implications of CSC Paradigm and Future Directions

The hypothesis that only CSCs are capable of reinitiating growth to form metastases in distant sites has fundamental clinical implications in terms of both prognosis and therapy since it provides an explanation of the limits to many current breast cancer treatments [87, 88]. In fact, the main goal of the current therapeutic strategies is represented by the “gold standard” of tumor shrinkage. However, if a tumor is maintained by a small subpopulation of CSCs that is constitutively resistant to therapeutic agents, tumor shrinkage results in the selective killing of the more differentiated, “nontumorigenic” cells that make up the bulk of the tumor, while leaving cancer stem cells viable and able to continue to maintain and/or reinitiate tumor growth on a metastatic site. Thus, current therapies fail to account for potential molecular and proliferative differences in the various subpopulations of tumor cells, and may be ineffective on the more aggressive and dangerous subgroup that constituted by CSCs. Since CSCs may constitute metastasis precursor cells, as suggested by the detection of disseminated tumor cells with a breast CSC phenotype in the bone marrow of breast cancer patients [83], it is of paramount importance to develop reliable diagnostic tools through the identification of additional and more specific markers for CSCs or even for niche cells, although this could be a difficult task due to the complexity of niche composition [89]. Difficult but not impossible, since recently Calabrese et al. [90] were able to visualize brain CSCs surviving in a vascular niche that secretes factors which promote their long-term growth and self-renewal. In addition, innovative technologies, using sensitive imaging techniques, have recently permitted real-time monitoring of CSC presence and viability as well as analysis of the angiogenic switch [91]. However, although this imaging technique can be extremely valuable in order to achieve a greater understanding of the biology of CSCs and their relationship with the stromal compartment, it cannot be used, at least at present, for intravital monitoring of such elusive cells in patients. Conversely, more clinically relevant imaging techniques, such as high-resolution magnetic resonance imaging (MRI) [92] and three-dimensional high-frequency ultrasound [93], are currently being developed for the study of CSCs in preclinical models. These techniques look promising for future clinical applications to determine prognosis, monitor therapeutic efficacy, and possibly affect therapies.

In addition to its impact on diagnosis and prognosis, the CSC hypothesis also has significant implications for the therapy of breast cancer. Since current therapies do not target the tumor-initiating cells effectively, as implied by the large number of patients who relapse after adjuvant chemotherapeutic and/or hormonal treatment [94], there is a need for therapeutic agents specifically directed against CSCs. These specific agents could be added to conventional cytotoxic drugs, designed to kill actively dividing cells, and be able to eradicate the metastatic disease. Thus, they would turn cancer, if detected at early stage, into a curable disease limited to the primary organ. Although we still know too little about the molecular features distinguishing CSCs from the bulk of tumor cells to develop a “smart drug”, the significant advances in the field indicate that CSCs could soon represent a really useful target.

## 10. Conclusions

Recent findings in breast biology have provided support for the CSC hypothesis, but researchers still face many challenges. First, attention should be paid to the accuracy of experimental methods for the isolation and propagation of CSCs derived from clinical samples, with a particular emphasis on cell culture environment (substrate, atmosphere, and medium) that has a critical role in standardizing the culture conditions for breast cancer progression studies [49]. Secondly, more accurate techniques should be used for the sphere formation assay, so as to determine a self-renewing capability sufficient to classify a cancer cell as a cancer stem cell, and to avoid conflicting results obtained by different groups. Moreover, it is necessary to pursue the clinical demonstration that CSCs can be used as a prognostic indicator of disease progression and to identify the mechanisms by which CSCs escape conventional therapies in order to develop new specific therapeutic approaches. Finally, there is the need to find the definitive evidence of the existence of CSCs and to identify the stroma-related factors that influence the development and spread of CSCs. Since CSCs have not yet been fully defined, their existence in breast cancer cannot conclusively be proven, and competitive hypotheses, which may explain some of the puzzling features of certain tumor cell populations, should be taken into account. The most exciting of these competitive hypotheses implies the reversible epithelial-to-mesenchymal transition, the developmental process in which epithelial cells acquire the migratory properties of mesenchymal cells. As shown in a very recent paper [95], the induction of the epithelial-mesenchymal transition could stimulate breast cells to adopt characteristics of stem cells. This suggests that CSCs are not distinct entities but rather tumor cells that transiently acquire stem cell-like properties as a consequence of an epithelial-mesenchymal transition. Undoubtedly, such a link between epithelial-mesenchymal transition and stem cell phenotype further fuels the debated question about the existence of CSCs and holds a number of interesting implications for the biology of epithelial cells, including the possibility that the stem cells of certain epithelial organs such as mammary glands may acquire many of the attributes of the mesenchymal cell state that confer them an increased tumorigenic potential.

The advent of new technologies, including gene expression profiling and proteomics, and the ability to apply them to small numbers of cells will probably help to solve such open questions.
